# Supplementary material for: Selection for Growth Performance in Oreochromis niloticus Across Different Aquatic Environments Using Growth Hormone Gene Polymorphisms
Source: Animals (Basel). 2025 Jul 16;15(14):2097. doi: 10.3390/ani15142097 (PMC12291795; doi:10.3390/ani15142097)
Supplement: Supplementary file 1 [file animals-15-02097-s001.zip › animals-3718114-supplementary.pdf]

Table S1. Results of significance testing for fixed effects on weight and length in Nile tilapia based on SNP and InDel variants in the *GH* gene.

SNP/InDel	Position	Trait	Fixed effect	Marker-*F*	*p*-value	Marker R^2	Error df
SNP_1	36	Weight	Population	9.80	0.218	0.75	154
SNP_1	36	Weight	Location	9.07	0.096	0.2	154
SNP_1	36	Weight	Age	6.79	0.187	0.33	154
SNP_1	36	Weight	Initial weight	9.52	0.179	0.16	154
SNP_1	36	Weight	Sex	4.54	0.300	0.08	153
SNP_1	36	Weight	SNP genotype	4.17	0.028*	3.1	154
SNP_1	36	Length	Population	6.08	0.020	0.7	153
SNP_1	36	Length	Location	3.46	0.100	0.97	154
SNP_1	36	Length	Age	7.11	0.160	1.86	153
SNP_1	36	Length	Initial weight	3.79	0.127	0.99	153
SNP_1	36	Length	Sex	2.98	0.117	0.96	154
SNP_1	36	Length	SNP genotype	4.3	0.006*	4.9	153
SNP_2	116	Weight	Population	7.96	0.118	0.33	153
SNP_2	116	Weight	Location	6.57	0.149	0.72	154
SNP_2	116	Weight	Age	2.65	0.075	0.4	154
SNP_2	116	Weight	Initial weight	1.18	0.151	0.01	152
SNP_2	116	Weight	Sex	1.83	0.150	0.84	154
SNP_2	116	Weight	SNP genotype	6.55	0.020	2.6	154
SNP_2	116	Length	Population	5.85	0.104	2.03	154
SNP_2	116	Length	Location	9.62	0.087	0.39	154
SNP_2	116	Length	Age	9.75	0.011	0.74	153
SNP_2	116	Length	Initial weight	6.90	0.064	0.19	154
SNP_2	116	Length	Sex	6.58	0.095	0.87	153
SNP_2	116	Length	SNP genotype	4.33	0.072*	3.5	154
SNP_3	134	Weight	Population	3.1	0.094	0.69	153
SNP_3	134	Weight	Location	1.07	0.198	0.38	153
SNP_3	134	Weight	Age	9.38	0.152	0.12	154
SNP_3	134	Weight	Initial weight	8.72	0.022	0.97	153
SNP_3	134	Weight	Sex	9.53	0.168	0.17	154
SNP_3	134	Weight	SNP genotype	2.12	0.042*	2.6	154
SNP_3	134	Length	Population	4.19	0.117	0.34	154
SNP_3	134	Length	Location	5.21	0.143	0.26	154
SNP_3	134	Length	Age	5.59	0.010	0.97	153
SNP_3	134	Length	Initial weight	5.76	0.087	0.45	154
SNP_3	134	Length	Sex	6.57	0.089	0.16	154
SNP_3	134	Length	SNP genotype	7.61	0.081	0.62	153
SNP_4	174	Weight	Population	1.53	0.094	0.24	154

*Associations were significant at $p \leq 0.05$ and were verified by the false discovery rate method.

Table S1. Continued

SNP_4	174	Weight	Location	3.57	0.066	0.47	154
SNP_4	174	Weight	Age	2.49	0.129	0.94	153
SNP_4	174	Weight	Initial weight	8.03	0.014	0.63	153
SNP_4	174	Weight	Sex	6.91	0.156	0.98	154
SNP_4	174	Weight	SNP genotype	1.46	0.045*	2.6	154
SNP_4	174	Length	Population	4.21	0.078	0.28	154
SNP_4	174	Length	Location	1.17	0.098	0.28	154
SNP_4	174	Length	Age	2.5	0.185	0.51	154
SNP_4	174	Length	Initial weight	3.44	0.143	0.74	154
SNP_4	174	Length	Sex	8.32	0.181	0.18	154
SNP_4	174	Length	SNP genotype	5.84	0.214	0.76	154
SNP_5	211	Weight	Population	7.67	0.133	0.98	154
SNP_5	211	Weight	Location	1.09	0.176	0.87	152
SNP_5	211	Weight	Age	3.74	0.800	0.3	154
SNP_5	211	Weight	Initial weight	3.38	0.185	1.5	153
SNP_5	211	Weight	Sex	6.31	0.093	0.7	153
SNP_5	211	Weight	SNP genotype	7.99	0.076	0.88	154
SNP_5	211	Length	Population	8.51	0.157	0.81	154
SNP_5	211	Length	Location	8.79	0.192	0.17	154
SNP_5	211	Length	Age	2.45	0.142	0.34	154
SNP_5	211	Length	Initial weight	3.17	0.138	0.66	153
SNP_5	211	Length	Sex	3.98	0.112	0.31	153
SNP_5	211	Length	SNP genotype	9.84	0.016*	3.8	153
SNP_6	240	Weight	Population	2.88	0.132	0.17	154
SNP_6	240	Weight	Location	1.91	0.067	0.93	154
SNP_6	240	Weight	Age	3.41	0.138	0.95	154
SNP_6	240	Weight	Initial weight	5.02	0.036	0.6	154
SNP_6	240	Weight	Sex	6.67	0.124	0.9	153
SNP_6	240	Weight	SNP genotype	2.05	0.351	0.93	154
SNP_6	240	Length	Population	2.39	0.124	0.79	154
SNP_6	240	Length	Location	3.14	0.095	0.94	154
SNP_6	240	Length	Age	3.13	0.038	0.81	153
SNP_6	240	Length	Initial weight	7.83	0.017	0.65	154
SNP_6	240	Length	Sex	2.83	0.157	0.51	154
SNP_6	240	Length	SNP genotype	6.59	0.015*	3.8	154
SNP_7	330	Weight	Population	4.20	0.130	0.36	153
SNP_7	330	Weight	Location	5.08	0.094	0.1	154
SNP_7	330	Weight	Age	3.01	0.011	0.19	154
SNP_7	330	Weight	Initial weight	3.35	0.071	0.79	154
SNP_7	330	Weight	Sex	2.86	0.196	0.39	154

*Associations were significant at $p \leq 0.05$ and were verified by the false discovery rate method.

Table S1. Continued

SNP_7	330	Weight	SNP genotype	9.94	0.231	0.56	153
SNP_7	330	Length	Population	5.90	0.194	0.29	154
SNP_7	330	Length	Location	8.67	0.900	0.28	153
SNP_7	330	Length	Age	7.09	0.400	0.83	153
SNP_7	330	Length	Initial weight	1.43	0.186	0.87	153
SNP_7	330	Length	Sex	9.14	0.056	0.5	154
SNP_7	330	Length	SNP genotype	4.30	0.008*	4.5	154
SNP_8	492	Weight	Population	3.56	0.179	0.26	154
SNP_8	492	Weight	Location	7.8	0.025	0.38	154
SNP_8	492	Weight	Age	8.01	0.152	0.18	153
SNP_8	492	Weight	Initial weight	7.03	0.173	0.59	153
SNP_8	492	Weight	Sex	4.52	0.194	0.77	154
SNP_8	492	Weight	SNP genotype	2.19	0.029*	3.1	154
SNP_8	492	Length	Population	5.58	0.196	0.23	154
SNP_8	492	Length	Location	2.19	0.184	0.7	154
SNP_8	492	Length	Age	8.28	0.064	0.62	153
SNP_8	492	Length	Initial weight	5.13	0.181	0.7	152
SNP_8	492	Length	Sex	9.93	0.104	0.36	154
SNP_8	492	Length	SNP genotype	8.71	0.007*	4.7	154
SNP_9	539	Weight	Population	3.6	0.134	0.56	154
SNP_9	539	Weight	Location	1.68	0.138	0.89	154
SNP_9	539	Weight	Age	3.85	0.200	0.32	154
SNP_9	539	Weight	Initial weight	7.04	0.033	0.58	154
SNP_9	539	Weight	Sex	5.54	0.144	0.2	154
SNP_9	539	Weight	SNP genotype	7.14	0.028*	3.1	154
SNP_9	539	Length	Population	9.97	0.092	0.73	153
SNP_9	539	Length	Location	4.74	0.141	0.63	154
SNP_9	539	Length	Age	4.42	0.188	0.28	154
SNP_9	539	Length	Initial weight	8.36	0.080	0.24	154
SNP_9	539	Length	Sex	9.15	0.017	0.14	154
SNP_9	539	Length	SNP genotype	8.75	0.017	0.19	154
InDel1	572	Weight	Population	5.33	0.019	0.88	153
InDel1	572	Weight	Location	2.51	0.122	0.35	154
InDel1	572	Weight	Age	1.16	0.017	0.28	153
InDel1	572	Weight	Initial weight	3.81	0.012	0.09	154
InDel1	572	Weight	Sex	8.62	0.100	0.77	154
InDel1	572	Weight	Genotype	9.9	0.230	0.41	154
InDel1	572	Length	Population	9.56	0.154	0.47	154
InDel1	572	Length	Location	1.58	0.034	0.900	154
InDel1	572	Length	Age	2.67	0.159	0.33	154

*Associations were significant at $p \leq 0.05$ and were verified by the false discovery rate method.

Table S1. Continued

InDel1	572	Length	Initial weight	8.39	0.166	0.33	154
InDel1	572	Length	Sex	5.12	0.124	0.3	153
InDel1	572	Length	Genotype	3.75	0.005*	4.9	154
InDel2	581	Weight	Population	7.47	0.191	0.800	154
InDel2	581	Weight	Location	9	0.069	0.700	153
InDel2	581	Weight	Age	5.6	0.022	0.97	154
InDel2	581	Weight	Initial weight	2.69	0.144	0.86	153
InDel2	581	Weight	Sex	2.07	0.091	0.95	154
InDel2	581	Weight	Genotype	1.10	0*	18.2	154
InDel2	581	Length	Population	7.08	0.037	0.75	153
InDel2	581	Length	Location	5.45	0.128	0.62	154
InDel2	581	Length	Age	3.59	0.066	0.18	154
InDel2	581	Length	Initial weight	7.42	0.039	0.48	154
InDel2	581	Length	Sex	3.8	0.155	0.81	154
InDel2	581	Length	Genotype	1.58	0*	14.8	152
InDel3	582	Weight	Population	3.12	0.141	0.31	154
InDel3	582	Weight	Location	1.47	0.046	0.03	153
InDel3	582	Weight	Age	9.11	0.155	0.44	153
InDel3	582	Weight	Initial weight	2.58	0.600	0.72	154
InDel3	582	Weight	Sex	5.91	0.160	0.42	154
InDel3	582	Weight	Genotype	3.67	0*	36	154
InDel3	582	Length	Population	8.57	0.072	0.15	154
InDel3	582	Length	Location	6.87	0.113	0.59	153
InDel3	582	Length	Age	2.27	0.128	0.48	154
InDel3	582	Length	Initial weight	3.9	0.020	0.86	154
InDel3	582	Length	Sex	6.53	0.117	0.87	153
InDel3	582	Length	Genotype	9.81	0*	23.4	153

*Associations were significant at $p \leq 0.05$ and were verified by the false discovery rate method.
